# Aryl Hydrocarbon Receptor Connects Inflammation to Breast Cancer

**DOI:** 10.3390/ijms21155264

**Published:** 2020-07-24

**Authors:** Tiziana Guarnieri

**Affiliations:** 1 Cell Physiology Lab, Dept. of Biological, Geological and Environmental Sciences (BiGeA), Alma Mater Studiorum Università di Bologna, 40126 Bologna, Italy; tiziana.guarnieri@unibo.it; Tel.: +39-051-2094253 or +39-339-4142317; 2 Interuniversity Consortium “Istituto Nazionale Biostrutture e Biosistemi” (INBB—Biostructures and Biosystems National Institute), 00136 Rome, Italy

**Keywords:** aryl hydrocarbon receptor, inflammation, breast cancer

## Abstract

Aryl hydrocarbon receptor (AhR), an evolutionary conserved transcription factor, is a pleiotropic signal transductor. Thanks to its promiscuous ligand binding domain, during the evolution of eukaryotic cells its developmental functions were integrated with biosensor functions. Its activation by a multitude of endogenous and exogenous molecules stimulates its participation in several pathways, some of which are linked to inflammation and breast cancer (BC). Over time, the study of this malignancy has led to the identification of several therapeutic targets in cancer cells. An intense area of study is dedicated to BC phenotypes lacking adequate targets. In this context, due to its high constitutive activation in BC, AhR is currently gaining more and more attention. In this review, I have considered its interactions with: 1. the immune system, whose dysregulation is a renowned cancer hallmark; 2. interleukin 6 (IL6) which is a pivotal inflammatory marker and is closely correlated to breast cancer risk; 3. NF-kB, another evolutionary conserved transcription factor, which plays a key role in immunoregulatory functions, inflammatory response and breast carcinogenesis; 4. kynurenine, a tryptophan-derived ligand that activates and bridges AhR to chronic inflammation and breast carcinogenesis. Overall, the data here presented form an interesting framework where AhR is an interesting connector between inflammation and BC.

## 1. Introduction

AhR, an environmentally sensitive transcription factor, is one of the more evolutionary conserved molecules in living cells. Since 550 million years ago, it has constantly been expressed in animal cells, but its exact physiological role remains elusive [1]. AhR was initially identified as a xenobiotic sensor and a key regulator of xenobiotics metabolism and persistent chemicals of concern, such as halogenated aromatic hydrocarbons (HAHs) and polycyclic aromatic hydrocarbons (PAHs). These compounds degrade very slowly in the environment, bio-accumulate in the food chain and are lipid soluble. Typically, they may be detected in human blood, adipose and breast tissue, where dichlorodiphenyldichloroethylene (DDE), the major metabolite of DDT (dichloro-diphenyl-trichloroethane), and PCBs (polychlorinated biphenyls) are the most prevalent contaminants [2]. In this scenario, 2,3,7,8-tetrachlorodibenzo-p-dioxin (TCDD) is the most infamous member of this class of environmental pollutants and one of exogenous best agonists of AhR [3]. Considering the evolutionary conservation, it makes sense to hypothesize that over time, biosensor functions have been added to its physiological role, which to this day remains elusive. As Mulero-Navarro and Fernandez Salguero recently pointed out, the role of AhR in systems homeostasis preceded its participation in xenobiotic sensing, as this function is lacking in invertebrates, where AhR homolog is a pivotal gene in the development of the nervous system, antennae, eyes and legs [4].

## 2. Structure, Physiology and Target Genes

**Figure 1:**
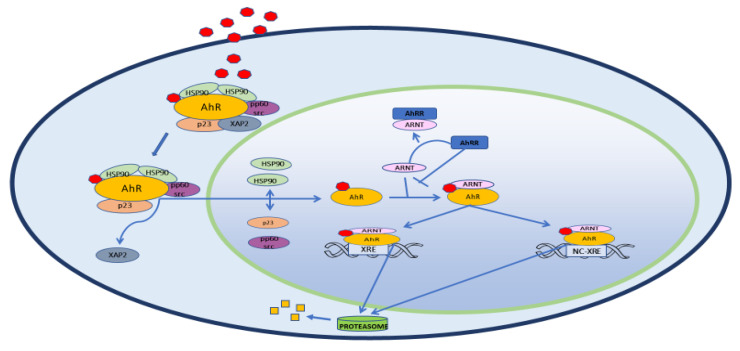
The Aryl hydrocarbon receptor (AhR) pathway. This transcription factor usually resides in the cytoplasm, where it associates with some inactivating molecules: two HSPs90s, one XAP2 or AIP, one HSP90 co-chaperone protein named p23, one pp60(c-src). Once it binds a ligand, XAP2 leaves the complex that moves to the nucleus. Here, the two HSP-90s, p23 and pp60-src drop AhR that binds to ARNT. This new complex is now ready to interact with XRE of genes like *CYP1A1*, *CYP1A2*, *CYP1B1* and *AHRR*, usually containing the sequences 5’-GCGTG-3′ or 5′-ACGTG-3′. AhR also targets NC-XREs, which bind AhR, while not containing the canonical consensus sequence. Just before the transcription starts, AhR moves from XREs, is translated out of the nucleus by XPO1 (not shown) and is degraded in the cytoplasm by 26S ubiquitin–proteasome. AhRR = Aryl Hydrocarbon Receptor Repressor; AIP = Immunophilin-like Ah Receptor-interacting protein); ARNT = Aryl Hydrocarbon Nuclear Translocator; CYP1A1 = Cytochrome P450 Family 1 Subfamily A Member 1; CYP1A2 = Cytochrome P450 Family 1 Subfamily A Member 2; CYP1B1 = Cytochrome P450 Family 1 Subfamily B Member 1; HSPs90 = Heat Shock Proteins 90; NC-XRE = Nonconsensus Response Element; p23 = Proteolytically Resistant 23-kDa protein; pp60(c-src) = Proto-oncogene tyrosine-protein kinase 60 (Sarcoma); XAP2 = Hepatitis B virus X-Associated Protein 2; XPO1 = Exportin 1; XRE = Xenobiotic Response Element.

**Figure 2:**
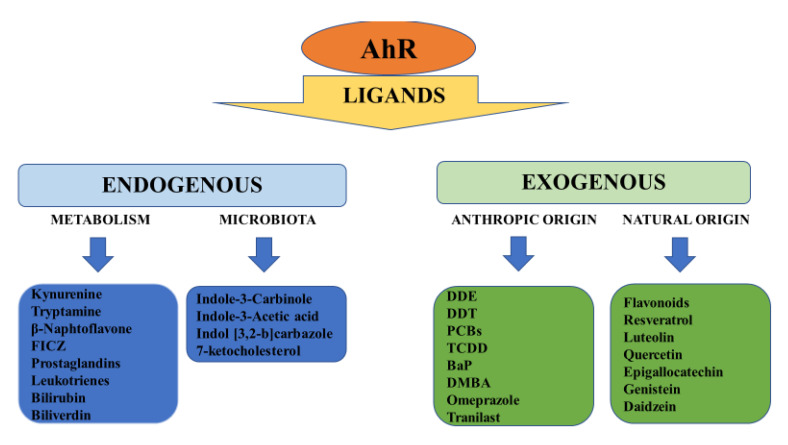
Endogenous and exogenous ligands of AhR. Endogenous ligands come from anabolism (kynurenine, tryptamine, β-Naphtoflavone, FICZ, prostaglandins, leukotrienes) and catabolism (bilirubin, biliverdin) of individuals and their commensal flora (microbiota: Indole-3-Carbinole; Indole-3-Acetic acid; Indol [3,2-b]carbazole; 7-ketocholesterol). Exogenous ligands can be manmade products which have a heterogeneous nature and uses (DDE, DDT, PCBs, TCDD, BaP, DMBA, Omeprazole Tranilast). Several exogenous ligands of natural origin are contained in plants and vegetable foods (Flavonoids, Resveratrol, Luteolin, Quercetin, Epigallocatechin, Genistein, Daidzein). DDE = dichlorodiphenyldichloroethylene; DDT = dichloro-diphenyl-trichloroethane; PCBs = polychlorinated biphenyls; TCDD = 2,3,7,8-tetrachlorodibenzo-p-dioxin; BaP = Benzo [a] pyrene; DMBA = 7,12-Dymethylbenz-[α]-anthracene.

In the cytoplasm, AhR forms a macromolecular inactivating complex with two heat shock proteins 90 (HSPs90), one XAP2 (hepatitis B virus X-Associated Protein 2 or AIP, Immunophilin-like Ah Receptor-interacting protein), one HSP90 co-chaperone protein named p23 and one pp60 src (Figure 1). After ligand binding, the AhR-inactivating complex is remodeled. XAP2 is released and, soon after, the complex moves to the nuclear compartment. Here, the two HSPs90 proteins, together with p23 and pp60 src, are replaced by AhR Nuclear Translocator (ARNT)/ hypoxia-induced factor β (HIF-1β). This heterodimer is the active form of AhR. It regulates the transcription of target genes through binding to the xenobiotic responsive elements (XREs) in their promoter containing the sequences 5′-GCGTG-3′ or 5′-ACGTG-3′ [5]. Once the transcription starts, AhR separates from XRE and is exported out of the nucleus by Exportin 1 (XPO1), also known as chromosome region maintenance 1 (CRM1). In the cytoplasm, it is inactivated by 26S ubiquitin–proteasome. Intriguingly, AhR self-regulates its nuclear activity as it activates the gene encoding for Aryl hydrocarbon Receptor Repressor (AhRR). This repressor competes with AhR for the binding with ARNT and forms the inactive heterodimer AhRR/ARNT. In this way, AhR lacks its partner that makes it transcriptionally active and its nuclear activity is hindered. Another target of AhR is the TCDD-inducible Poly ADP-Ribose Polymerase (*TIPARP*) gene, which negatively regulates *AR* expression and thus AhR levels [6]. These negative feedback mechanisms, together with the nuclear exportation and ubiquitin-proteasome-mediated degradation of AhR, regulate the transcription of AhR-responsive genes [7]. Back in 1983, Israel and Whitlock showed that cytochrome P_1_-450 genes (*CYP450*, class 1A (1 and 2)*)*, encoding enzymatic metalloproteins involved in endogenous and exogenous substrates transformation, are targeted from “the TCDD receptor”, later identified as the AhR [8]. In the year 2000, Nebert group described four additional AhR target genes: 1. *ALDH3A1* (Aldehyde dehydrogenase family 3, subfamily 1); 2. *GSTA1* (Glutathione S-transferase, alpha 1); 3. *NQO1*, (NAD(P)H dehydrogenase quinone 1); 4. *UGT1A6* (UDP glucuronosyltransferase family 1 member A6) [9]. Together with *CYP1A1 AND CYP1A2* genes, they form the so-called “AhR gene battery”, which controls the cell cycle and the initiation of the apoptotic cascade. Later, other groups confirmed and extended the list of AhR-responsive genes which are involved in different steps of cell lifecycle and metabolism. These include also Phase III transporters, as the Proteins 2 and 3 associated to multidrug resistance (*MRP2-MRP3*), the organic anionic and cationic transport proteins (*OATP and OCTP*), the breast cancer resistance proteins (*BCRP*) [10,11,12,13]. More recently, it has been suggested that AhR participates in pluripotency and stemness regulation through a possible link with transposable elements [14]. Alternative, “unorthodox” signaling has been described in the last few years [15], as it has been observed that some AhR-responsive genes do not contain the XREs. This is the case for plasminogen activator inhibitor-1 (*PAI-1*), encoding a fibrinolysis inhibitor which has been connected to inflammatory endothelial fibrosis. The *PAI-1* promoter is not a classical XRE, but is responsive to TCDD, one of the more potent exogenous agonists of AhR binding. In fact, Wright group described a nonconsensus XRE (NC-XRE) in *PAI-1* promoter which interacts with AhR alone when it is associated to Kruppel-like factor 6 (KLF6) [16]. AhR dimerizes with the KLF family member KLF6 and binds to a novel nonconsensus XRE (NC-XRE) in the promoter of target genes after TCDD exposition. NC-XRE and XRE have no sequence homology and interact with different proteins, this suggesting that AhR also has different targets. In particular, they demonstrated that the complex AhR/KLF6 is of pivotal importance in the control of cell cycle, as it regulates the expression of p21Cip1, or, cyclin-dependent kinase inhibitor 1A (CDKN1A), which inhibits the cell cycle and regulates cell metastasis by switching between invasion and proliferation [17]. Considering these data, it is reasonable to hypothesize that originally AhR had a checkpoint role in the cellular metabolism and that, over time, AhR has acquired the ability to bind to a multitude of molecules, both exogenous and endogenous. Over time, it has been demonstrated that, due to its promiscuous binding site, AhR is responsive not only to exogenous, but also to a variety of natural molecules, among which some derivatives of arachidonic acid as prostaglandins and leukotrienes, lipotoxin A 4 and 7-ketocholesterol [18], the hemoglobin catabolites bilirubin and biliverdin, the tryptophan products kynurenine (Kyn), tryptamine and 6-formylindolo[3,2-b] carbazole (FICZ), some indole metabolites derived from diet and from host bacteria metabolism, such as Indole-3-acetic acid (IAA) and Indol [3,2-b]carbazole (ICZ) (Figure 2) [19,20]. The list of possible AhR ligands is constantly growing and some of these compounds are now included into the category of selective AhR modulators (SAhRMs), that bind AhR with low to medium affinity. Interestingly, they can behave as an agonist, antagonist or a mixed agonist/antagonist, depending on the metabolic set-up of the organism with which they come into contact [21]. Most information about the role of AhR in organ and system physiology comes from multiple systemic abnormalities described in AhR^−^/AhR^−^ mice. Obviously, these rodents are not sensitive to TCDD [22] and are also smaller in size than AhR^+^/AhR^+^ mice. They exhibit a spectrum of anomalies that includes reduced fertility, portal tract fibrosis, hepatocyte microvesicular fatty metamorphosis, cardiac hypertrophy, epidermal hyperplasia, T cell deficiency in the spleen [23] and altered circadian rhythms [24].

## 3. AhR and Inflammation

From 2000 onwards, various groups have reported a connection between AhR and inflammatory phenomena in different experimental models. In 2002, Hennig group showed that in endothelial cells, the PCB-mediated activation of AhR promotes inflammatory atherosclerotic phenomena, passing through the transcriptional activation of NF-κB and the increase in IL6 production [25]. NF-κB/AhR interactions in inflammation were also described in detail by Tian and colleagues [26] and, in the same period, an interesting review by Dalton was focused on the connection between AhR, inflammatory signaling and oxidative stress [27]. Over the years, AhR’s pathway has been associated with various inflammatory markers, including cyclooxygenase-2 (COX2), tumor necrosis factor α (TNFα), matrix metalloproteinases (MMPs) [28], early growth response 1 (EGR1) [29], prostaglandin E _2_ (PGE_2_), microsomal PGE_2_ synthase (mPGE_2_S) [30], NF-κB component RelB [31], RelA [32,33] inducible nitric oxide synthase (iNOs) [34], interleukin 8 (IL8) [35]. 

### 3.1. AhR in the Immune System 

Considering the biosensor role of AhR, it is not surprising that most immune cells express AhR [36]. Accumulating evidence highlights that AhR has a role in the regulation of the immune system and inflammation. As early as the 1980s, Poland observed an immunosuppressive effect after TCDD exposure [37]. Consistently, in 1991 Holsapple and colleagues [38] described a sort of “immune system failure” in TCDD-exposed mice, where the functions of thymus and other immune organs were profoundly impaired and circulating lymphocytes lowered considerably. In 1998, an in vitro research of Sulentic research group [39] evidenced that TCDD exposure activates murine B cells, induces the expression of *AHR* gene and protein and is followed by the suppression of IgM secretion. Interestingly, at the threshold of 2000, AhR was “suspected” to mediate TCDD biological effects. In 2003, Doi and colleagues [40] demonstrated that when mice primary mature T cells and B lymphocytes are exposed to TCDD, AhR is activated and induces the *CYP1A1* gene, thus confirming the interaction between AhR and the immune system. AhR participates to the commitment of T cells and Th_17_ cells development and differentiation [41]. In addition, Quintana group highlighted that AhR influences Th_17_ and T_reg_ cells differentiation with ligand-dependent basis [42]. As an example, it has been observed that TCDD is able to activate T_reg_ cells, thus limiting autoimmune responses. FICZ, a potent endogenous activator of AhR which derives from tryptophan metabolism, halts Treg metabolism and promotes the development of Th_17_ cells. This favors autoimmune processes. In vivo observation in AhR^−^/^−^ mice confirms the leading, but not yet completely defined role of AhR in regulating the immune system. In fact, all three strains of AhR knockout mice are affected by hepatic and vascular defects, but only one strain shows immune system abnormalities represented by T cells deficiency in their spleens [43]. 

### 3.2. AhR and NF-ΚB

Interestingly, some defects observed in the immune organs following TCDD exposition are controlled by NF-κB or Rel proteins. NF-κB is a pleiotropic dimeric transcription factor which is expressed in most cell types. In physiologic conditions, it controls proliferation, cellular growth, differentiation and apoptosis. NF-κB is currently considered one of the most influential factors in immune reactions and inflammation control. It is inducible from many pro-inflammatory ligands such as inflammatory cytokines, reactive oxygen species bacteria, stress inducers and a large array of drugs [44]. In turn, it influences the expression of a plethora of target genes, among which inflammatory cytokines. As early as 1999, Tian and colleagues [45] assessed in liver and kidney cell lines the physical association between AhR and RelA, the p65 subunit of NF-κB. This association hesitates in their reciprocal functional repression. They hypothesized that when AhR is activated by TCDD it associates with and antagonizes RelA, thus explaining the immunosuppressive effect of TCDD. In this study, they evidenced the leading role of NF-κB in modulating the pathway of AhR, which is activated by TCDD administration and suppressed by TNFα-induced activation of NF-κB. In 2004, Sulentic group [46] examined the 3’alpha Ig heavy chain enhancer, a transcriptional regulatory element in Ig heavy chain genes. This enhancer has a regulatory domain called hs4, which contains a kappaB motif overlapping an XRE-like site. On this basis, they demonstrated that AhR and NF-κB/Rel proteins act cooperatively and converge at the XRE and KappaB motif. Here, they modulate the transcription of the hs4 fragment of the 3’alpha immunoglobulin heavy chain enhancer. This cooperation is also the mechanism which sustains the inhibition of the 3’Igh regulatory region of the murine immunoglobulin (Ig) heavy chain gene by TCDD, which results in the decrease of Ig expression and thus in circulating antibody [47]. The interaction between AhR and NF-κB is even more evident in the inflammatory response. More generally, a consistent number of inflammation-related genes possess putative XREs [48,49] which are the target of AhR in immune and non-ddeimmune cells. Of note, NF-κB is hub of many inflammatory pathways, which converge, diverge and connect with this molecule. In most, if not all cancer cells, NF-κB is activated as the result of mutations, epigenetic alteration or, more likely, the presence of proinflammatory cytokines, such as IL6 and TNFα, which are secreted in the tumoral inflammatory microenvironment. Interestingly, proinflammatory factors are secreted not only from inflammatory cells, but also from inflamed stromal and tumoral cells. In the last ten years, several papers about the interaction between AhR and NF-κB in inflamed or tumoral cells have been published. This transcription factor is a pivotal element involved in inflammation, in the modulation of immunity—both innate and adaptive—and in normal/tumoral proliferation. In 2008, Vogel and Matsumura [50] expanded the data about the crosstalk between AhR and NF-κB, as they described an AhR/RelB dimer. Contrary to RelA, which has a repressive action on AhR, the RelB subunit has a collaborative behavior. Rel B is generated by Lymphotoxin (LT)-α_1_β_2_ and selected ligands of the tumor necrosis factor receptor (TNFR) through the activation of the “non canonical” NF-κB pathway. After stimulus starting from the processing of p100 protein, two kinases, NIK (NF-κB-Inducing Kinase) and IKKα (Inhibitor Kappa B Kinase α) originate RelB, also called p52. This alternative pathway is delayed with respect to the one that generates RelA, which accounts for rapid responses. Intriguingly, after nuclear translation, both RelA/p65 NF-κB and RelB/p52 NF-κB dimers bind both to XREs, both to NF-κB targets. The regulatory activity of these dimers converges in a complex framework where, until recently, it was believed that RelA controls inflammatory responses and innate immunity, while RelB regulates B cell maturation and differentiation and the development of secondary lymphoid organs. The current view integrates their functions in a comprehensive design where NF-κB and AhR are partners in some inflammatory conditions [51]. Their relationship is dependent on the tissue and the metabolic context. In breast cancer cell lines and in tumor breast cells, particularly those with a “triple negative” (TNBC, triple negative breast cancer) phenotype (so defined because tumor cells do not express the estrogen receptor (ER), progesterone receptor (PR) and the HER2 isoform of the epithelial growth factor receptor, EGFR [52]), AhR cooperates with NF-κB. This interaction takes concrete form through the formation of an AhR/RelB dimer. AhR/RelB binds both XREs and NF-κB responsive elements, thus activating the transcription of AhR and NF-κB responsive genes supporting cell inflammation and proliferation [50].

## 4. AhR and Cancer

More generally, inflammation is an ascertained risk factor for the onset of a plethora of pathologies, among which cancer [53,54,55]. Here, a frequently emerging role in the modulation of the inflammatory state is emerging for AhR. In 2003, Jensen and colleagues [56] described the halting of IL6 expression by two prototypic AhR agonists, 7,12-dimethylbenz [a]anthracene (DMBA) and TCDD in bone marrow cells treated with the pro-inflammatory bacterial lipopolysaccharide (LPS). More recently, in HepG2 cells, a Stobbe-Maicherski study [57] evidenced the modulation of the expression and activity of AhR by oncostatin M (OSM). This cytokine is member of the IL-6 family and regulates hepatic cell growth and proliferation. In inflammatory contexts, it also stimulates the transcription of *AHR*, which makes cells reactive to xenobiotic and environmental pollutants. To date, conflicting evidence connects AhR to cancer onset, promotion and progression. In our opinion, a multiplicity of factors determines the possible involvement of AhR in tumors. First, as anticipated, an inflamed microenvironment can promote the pro-tumoral potential of AhR factor. As we argumented in a recent paper [58], AhR is involved in inflammatory phenomena both as cause and effect of pre-existing inflammations. A large body of literature has shown that, upon binding exogenous molecules, as HAHs and PAH, AhR can endow inflammatory phenomena that sustain the malignant transformation of normal cells [59].

### 4.1. AhR and Breast Cancer

The connection between inflamed phenotype and neoplasia is now evident in BC [60,61]. BC is the most common invasive malignancy among women in the industrialized world. It is a complex, multifactorial and extremely heterogeneous disease. Genetic factors account for up to 10% of all BCs. Among inherited mutations, *BRCA1 e BRCA2* (BReast CAncer) suppressor genes are the most involved in BC susceptibility. BRCA1 interacts both at transcriptional and post-transcriptional level with estrogens pathway, in order to limit their positive effects on proliferation of mammary tissues. The lack of this control is a well-known risk factor for TNBC occurrence [62] and this can occur due to *BRCA1* epigenetic mutations, among which AhR-instigated hypermethylation [63].

Molecular classification of BCs includes luminal subtypes, which are ER/PR-positive and HER2 and Ki-67 (a proliferative marker protein)-negative (Luminal A), or HER2-positive/negative with high Ki-67 levels (Luminal B). TNBCs, which are more frequent in young, Mediterranean/black females, are ER/PR-negative and HER2-negative. This subtype is commonly associated with inactivating mutations in *BRCA1* and *BRCA2* genes. Lastly, HER-2-enriched BC subtype is ER/PR -negative and HER2 -positive [64]. HER2 -positive tumors can be almost successfully treated with anti-HER2 chemotherapeutics such as trastuzumab, lapatinib and neratinib. For a long time, hormone-positive tumors have been treated with antagonists (tamoxifen) of their steroid receptors binding or aromatase enzymes inhibitors, which halt their biosynthesis. These drugs are often associated with CDK 4/6 (cyclin-dependent kinase 4/6) inhibitors to sensitize hormone receptor-positive, HER2 negative metastatic breast tumors to chemotherapy. Another therapeutic option are drugs inhibiting the enzyme Poly ADP ribose polymerase (PARP), which repairs single strand breaks in DNA. Obviously, its inhibition leads to tumoral cell death [65].

Some interesting data report the onset of more aggressive, chemotherapy-resistant BCphenotypes in ER-positive cells residing in inflamed microenvironments [66,67]. Early studies suggest an association between some AhR ligands exposition and BC incidence [68,69]. It has also been suggested that, in inflammatory conditions, AhR sustains the pro-tumoral potential of some cytokines, such as IL6 [70].

Accordingly, the coexistence of AhR activation and inflammatory conditions are recurring elements in mammary tumors. In 1997, Sovak group [71] demonstrated a highly constitutive NF-κB expression in human breast primary and breast cancer cells and some years later, the same group reported that, together with AhR, NF-κB participates in the tumoral transformation of mammary cells. They showed that in murine normal mammary cells treated with DMBA and in human non-transformed breast cells treated with DMBA or benzo[a]pyrene (B[a]P), the activity of NF-κB (p65/p50) increases during tumoral transformation [72]. Consistently, if non-transformed breast cells MCF 10F (ER^−^, PR^−^, and HER2^−^) are treated with DMBA or B[a]P, AhR and the NF-κB subunit RelA integrate their functions to activate the transcription of the proto-oncogene MYC and the expression of the protein c-myc, a master regulator in cell proliferation and tumoral transformation [73]. In addition, in DMBA-induced murine mammary tumors, it has been described that there is a high expression of AhR, the oncogenes cyclin D1 and c-myc, in association with NF-κB and Wnt signalling [74]. Interestingly, in BC, AhR synergizes with NF-κB and modulates the metabolism of IL6 [75].

IL6 is a multifunctional cytokine, ranging from 22 and 30 kDa. Its expression is regulated from NF-κB and is involved in immune function, hemopoiesis, acute phase response and inflammation. Depending on its localization and concentration, the role of IL6 ranges from physiology to pathology [76], especially in mammary tissue [60]. Here, together with its downstream factor STAT3, it stimulates cell proliferation and migration during ontogenesis, while it is involved in gland remodeling during aging [77]. In breast, IL6 levels are further progressively increasing with age due to the decrease of sex steroids, which exert during life a feedback mechanism control on the cytokine [78]. When IL6 concentration overcomes normal levels, an inflammatory process takes place, being the more detrimental to cell functions the higher its levels and the duration of inflammation. Consistently, high serum and tissues levels of IL6 have been found in TNBC [79,80]. Consequently, IL6-driven inflammation is considered one of the most influential risk factors in TNBC.

### 4.2. AhR and TNBC

This type of BC affects women in all age groups. It is a highly aggressive neoplasia whose cells, as anticipated above, do not express ER, PR and HER2. Therefore, it has fewer options for targeted treatment, as hormone therapy and HER2 drugs cannot be used. For this reason, its outcome is not favorable. Over the last 10 years, the urgency to identify alternative therapeutic options for TNBC has fueled the selection and the screening of selected molecular targets, some of them belonging to the AhR pathway.

Recently, high expressions of AhR have been described by Romagnolo group in some TNBC cell lines [63]. TNBC cells are characterized from high proliferation/metastatic grade and invasive ability. The coexistence of high expression of IL6 and AhR in this phenotype led many authors to test the possibility of a mutual interplay. So far, the possible connection between inflammation and AhR pathway has been studied in different cell types and models, where a combination of AhR-activating molecules and inflammatory agents has been used. In 2008, Hollingshead group [81] obtained an inflammatory state in MCF-7 breast and ECC-1 endocervical cancer cells, through the administration of IL-1β or phorbol 12-myristate 13-acetate (PMA), in combination with TCDD-induced activation of AhR. This treatment resulted in a prolonged stimulation of IL6 expression, which was also sustained by Rel A, the p65 subunit of NF-κB. In 2011, Opitz and colleagues [82] proved that AhR is expressed and constitutively active in gliomas, due to the presence of tryptophan-2,3-dioxygenase (TDO). TDO and IDO (indoleamine-2,3-dioxygenase) are the rate-limiting enzyme of a pathway which starts from tryptophan (trp) and produces Kyn, an endogenous ligand of AhR. Kyn acts on two fronts: on the one hand it strongly inhibits the immune response and, on the other, it binds to AhR and promotes cell growth and motility. These data match with those reported in TNBC by Novikov group, which in 2016 described a positive amplification loop involving TDO and its products Kyn and xanthurenic acid (XA), both AhR ligands [83]. The starting point of Novikov’s research was the overexpression of *AHR* and *TDO2* in BC. In primary human TNBC, the *TDO2* expression level is higher than in ER^+^ tumors and adjacent normal tissue. In ER^−^/PR^−^/Her2^−^ cell lines, the amount of Kyn and XA is so high that they constantly stimulate the expression of AhR. The overexpression of AhR is paralleled by a similar increase in cell migration, which is representative of the metastatic process and the aggressiveness of BC type. Intriguingly, in TNBC cell lines, the decrease of TDO2 mRNA expression after *AHR* knockdown demonstrated that AhR participates in the *TDO2* expression control mechanism. So, they depicted an amplification loop where AhR is the hub. Interestingly, both exogenous and endogenous ligands can modulate AhR functionality, both positively and negatively [84,85]. As anticipated, trp depletion results in immunosuppression and chronic inflammation. Consistently, in 2013, Litzenburger demonstrated the key role of AhR in an autocrine signaling loop expressing IDO [86]. IDO is expressed in several tumoral forms and cell lines, among which the breast MCF-7. Here, it transforms trp in Kyn. Once Kyn is produced, it binds AhR, which moves to the nucleus and activates the expression of IL6 gene. Then, IL6 is secreted and, once released into the intercellular microenvironment, it binds to its receptor IL6R. IL6R activation gives rise to a signaling cascade that includes the STAT3 transcription factor. STAT3 is the element that completes this loop, as it moves into the nucleus and binds the response element in the gene encoding for IDO, which produces Kyn. In this loop, the immunosuppressive mechanism, driven by trp transformation in Kyn, is connected to the inflammatory pathway, driven by IL6. The interplay between AhR, inflammatory pathway and immunomodulation in BC was recently confirmed by Vacher research group [87]. They assessed the expression of AHR both in ERα-positive or ERα-negative human breast tumors and found in ER-positive cells a correlation between AHR expression, metastasis occurrence and related markers (MMP1, MMP2), and plasminogen activator urokinase (PLAU). Consistently, the expression of *AHRR*, which encodes the transcription factor that competes with AhR for ARNT binding, is protective against metastatization. In addition, the higher the expression of AhR, the greater is the expression of genes encoding for inflammatory factors, trp metabolism, invasive behavior and insulin growth factor (IGF) signaling. According to this data, the administration of the AhR agonists TCDD or BaP (Benzo [a] pyrene) induces the expression of the pro-inflammatory cytokines IL1β and IL6 in ER-negativecells. Interestingly, in ER-negative breast tumors, a strong association between *AHR* overexpression and BRCA1 mRNA exists, thus indicating a likely participation of AhR in DNA repair.

These studies evidence the direct connection between inflammation and the AhR pathway in breast cells and tumors. They are also closely connected with previous studies of Goode and colleagues [88]. They proposed that in TNBC tumors and cell lines, AhR expression is directly proportional to tumoral aggressive behavior, both in vivo and in vitro. In MDA-MB231, a TNBC cell line, they observed the attenuation of the malignant phenotype after *AHR* knockdown. Considering these premises, AhR inhibition could be a promising therapeutic target in TNBC. This result can be obtained in vivo by the administration of selected antagonists, among which genistein. The dietary intake of this isoflavone had been shown to inhibit AhR interaction with BRCA1 exon 1 in mice mammary tissue and AhR-driven hypermethylation of CpG in *BRCA1* gene. These results are consistent with those obtained by the administration of the synthetic α-naphthoflavone and the natural flavonoid Galangin in MCF7 cells. The antiproliferative properties of these molecules have long been known and are supported by data identifying them as AhR modulators in TNBC [89]. Interestingly, galangin and the natural isoflavone genistein are also known for their anti-inflammatory properties [90,91].

As documented, inflammation is a recognized risk factor for several tumors. In BC, some risk factors are intrinsic: sex and race, as females are more affected than males with a ratio of 70 (black men) and 100 (white men) to 1 woman; age, as its occurrence is higher in women over 50 years of age and in older men; genotype, as some mutations, i.e., involving BRCA1 and BRCA2 are associated with an increased occurrence and worse prognosis. Unlike intrinsic risk factors, extrinsic risk factors can be modified, as they are related to lifestyle. Smoking, physical activity, sleep deprivation eating and consuming alcohol and high body mass index can be corrected focusing on primary prevention [92]. Interestingly, both intrinsic and extrinsic risk factors influence the individual inflammatory state, which can be reduced by changing unhealthy habits and taking anti-inflammatory drugs [93]. In this context, AhR could be a therapeutically interesting target as selected AhR ligands have been demonstrated to exert anti-inflammatory and antitumoral effects in in vitro and in vivo experimental models of BC. In this context, it is essential to understand the exact role that AhR plays in the different inflammatory pathways which develop in BC. This could be of relevant importance in TNBC, which yet, lacks specific therapeutic targets. Differently from other types of BC, in TNBC AhR is frequently overexpressed. It has a proinflammatory role, as it interacts with several inflammatory pathways, which, in some cases, develop thanks to an active interaction between cancer cells and stroma [94].

AhR has a key role in the initiation and promotion of severe TNBC. Its overexpression is linked to the inhibition of *BRCA1* gene by means of hypermethylation [95] which is a highly predisposing factor to TNBC, as BRCA1 is a tumor-suppressing gene. Moreover, in breast, BRCA1 limits aromatase expression, and thus estrogens production. So, the net result of an AhR-induced *BRCA1* inhibition is the stimulation of aromatase and E2 (estradiol) increase in tumor cells, which sustains cell proliferation. Consistently, in BC cell lines treated with AhR agonists, the activation of the aromatase gene and an increase in E2 production were described. Interestingly, IL-6 mRNA and TNF mRNA are also strongly induced while *BRCA1* mRNA decreases [96]. A pivotal point in TNBC occurrence is AhR interaction with the ER pathway. In fact, it has been known for about 15 years that AhR inhibits ER-dependent signaling through the recruiting of the proteasome complex [97,98]. An additional piece of evidence is that BRCA1 transactivates *ESR1* gene, which encodes for ERα. Thus, the contradictory effect of activated AhR on ERα (inhibition of signaling) and E2 expression (increase) is probably related to the AhR-induced inhibition of *BRCA1* by means of hypermethylation. This mechanism could also be the reason for the failure of clinical trials testing the effectiveness of AhR agonists in BC chemotherapies. From an integrated perspective, this interplay is of pivotal importance, because it could be modulated with appropriate dietary strategies. As Donovan and colleague pointed out in a recent review [88], the *antagonism* towards AhR could rescue *BRCA1* functionality and ERα sensitivity, which is necessary for successful chemotherapies. As already stated, the overactivation of AhR in TNBC is strictly correlated with the development and maintenance of inflammation [87]. Therefore, the administration of AhR antagonists, both in combination with chemotherapy [99] and within a balanced diet, could combine the containment of inflammation with common cytostatic therapies that are used to fight this type of cancer. Their habitual consumption may also be part of a preventive strategy, especially when there is a family history of BC with *BRCA1* gene inhibiting mutations.

## 5. Conclusions

**Figure 3:**
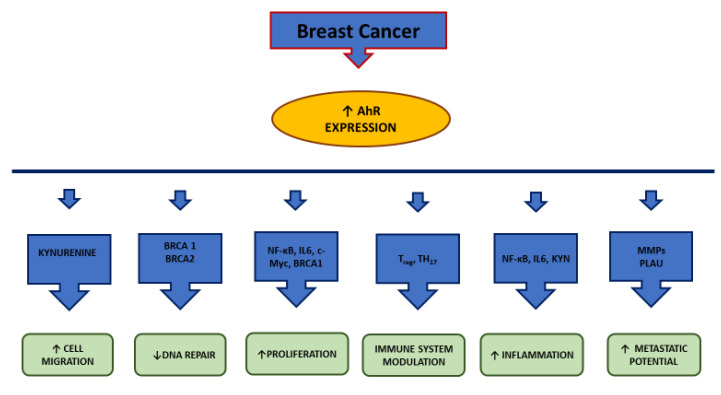
AhR pathway interactions in cancer breast cells. AhR is usually overexpressed in cancer breast cells. Here, it correlates with several markers of: 1. proliferation (NF-κB, IL6, c-Myc, BRCA1); 2. DNA repair (BRCA genes); 3. cell migration (Kyn) 4. metastatic behavior (MMPs and PLAU); 5. immune system (Th_17_ and T_reg_ cells); 6. Inflammation (NF-κB, IL6, Kyn); BRCA = BReast CAncer gene; Kyn = Kynurenine; c-Myc = Avian myelocytomatosis virus oncogene cellular homolog; IL-6 = interleukin 6; MMP = matrix metalloproteinase; NF-kB = nuclear factor kappa-light-chain-enhancer of activated B cells; PLAU = Plasminogen Activator, Urokinase *(gene);* ↑ = increase; ↓ = decrease.

In conclusion, it appears evident that there is a clear link between AhR pathway, inflammation and BC, particularly triple negative (Figure 3). Since inflammation is one of the factors that favors and supports tumor transformation through several factors, among which AhR, it is reasonable to include AhR among the targets of a combined anti-inflammatory/antitumoral therapy.

## Abbreviations

AhR	Aryl hydrocarbon receptor
AhRR	Aryl hydrocarbon receptor repressor
AIP	Immunophilin-like Ah receptor-interacting protein)
Aldh3a1	Aldehyde dehydrogenase family 3, subfamily 1
ARNT	Aryl hydrocarbon nuclear translocator
BaP	Benzo[a]pyrene
BC	Breast cancer
*BCRP*	Breast cancer resistance proteins
BRCA	BReast CAncer gene
CDK 4/6	Cyclin-dependent kinase 4/6
CDKN1A	Cyclin-dependent kinase inhibitor 1A
c-Myc	Avian Myelocytomatosis virus oncogene cellular homolog
COX2	Cyclooxygenase-2
CpG	C phosphate G
CRM1	Chromosomal Maintenance 1
CYP1A1	Cytochrome P450 family 1 subfamily A member 1
CYP1A2	Cytochrome P450 family 1 subfamily A member 2
CYP1B1	Cytochrome P450 family 1 subfamily B member 1
DDT	Dichlorodiphenyltrichloroethane
DMBA	Dimethyl-benz(a)anthracene
E2	Estradiol
ECC-1	endocervical cancer cells
EGFR	Epithelial growth factor receptor
EGR1	Early growth response 1
ESR1	Estrogen receptor gene
ER	Estrogen receptor
FICZ	6-formylindolo[3,2-b] carbazole
Gstα1	Glutathione S-transferase, alpha 1
HAHs	Halogenated aromatic hydrocarbons
HepG2	Hepatoma G2
HER2	Human epidermal growth factor receptor 2
HIF-1β	Hypoxia-induced factor β
HSPs90	Heat shock proteins 90
IAA	Indole-3-acetic acid
ICZ	Indol [3,2-b]carbazole
IDO	Indoleamine-2,3-dioxygenase
IGF	Insulin growth factor
IKKα	Inhibitor kappa B kinase α
IL1β	Interleukin 1β
IL-6	Interleukin 6
IL-8	interleukin-8
iNO	induced nitric oxide oxide
KLF6	Kruppel-like factor 6
Kyn	Kynurenine
LPS	Lipopolysaccharide
LT-α_1_β_2_	Lymphotoxin-α_1_β_2_
MCF 10F	Michigan Cancer Foundation 10 Floating
MDA-MB231	M.D. Anderson - Metastasis Breast 231
MMP	Matrix metalloproteinase
mPGE_2_S	microsomal PGE_2_ synthase
*MPR2*	Multidrug resistance protein 2
*MPR3*	Multidrug resistance protein 3
mRNA	Messenger ribonucleic acid
NC-XREs	Nonconsensus response elements
NF-kB	Nuclear Factor kappa-light-chain-enhancer of activated B cells
NIK	NF-κB-inducing kinase
Nqo1	NAD(P)H dehydrogenase quinone 1
OATP	Anionic transport proteins
*OCTP*	Organic cationic transport proteins
OSM	OncoStatin M
p23	Proteolytically resistant 23-kDa protein
PAHs	Polycyclic aromatic hydrocarbons
PAI-1	plasminogen activator inhibitor-1
PARP	Poly ADP ribose polymerase
PCBs	Polychlorinated biphenyls
PGE_2_	Prostaglandin E_2_
PLAU	Plasminogen activator urokinase
PMA	Phorbol 12-myristate 13-acetate
pp60(c-src)	Proto-oncogene tyrosine-protein kinase 60 (Sarcoma)
PR	Progesterone receptor
RelA	V-Rel reticuloendotheliosis viral oncogene homolog A
RelB	V-Rel Reticuloendotheliosis viral oncogene homolog B
SAhRMs	Selective AhR modulators
STAT3	Signal transducer and activator of transcription 3
TCDD	2,3,7,8-Tetrachlorodibenzo-p-dioxin
TDO	Tryptophan-2,3-dioxygenase
TiPARP	TCDD-inducible poly ADP-ribose polymerase
TNBC	Triple negative breast cancer
TNFR	Tumor necrosis factor receptor
TNFα	Tumor necrosis factor-α
Ugt1a6	UDP glucuronosyltransferase family 1 member A6
Wnt	Wingless/Integrated
XA	Xanthurenic acid
XAP2	Hepatitis B virus X-associated protein 2
XPO1	Exportin 1
XREs	Xenobiotic response elements
